# Functional performance improvement data and patent sets for 30 technology domains with measurements of patent centrality and estimations of the improvement rate

**DOI:** 10.1016/j.dib.2020.106257

**Published:** 2020-09-02

**Authors:** Giorgio Triulzi, Christopher L. Magee

**Affiliations:** a Universidad de los Andes, School of Management, Colombia; Massachusetts Institute of Technology, Institute for Data, Systems and Society, United States; b Massachusetts Institute of Technology, Institute for Data, Systems and Society and International Design Center, United States

**Keywords:** Performance curves, Moore's law, Improvement rates, Technological change, Technology dynamics, Patent centrality

## Abstract

This article accompanies the study presented in Triulzi et al. (2020) [1]. It briefly describes and makes available the data on functional performance for 30 technology domains, their patent sets, the measurement of patent centrality and the method to estimate the yearly technology performance improvement rate (TIR) that underly that study. Some of this data (performance time series and the lists of patents for 28 domains) has been collected by other authors for previous studies but were previously unavailable to the public. Measurements of patent centrality and other patent-based indicators for the 30 domains, and for 5.259.906 utility patents granted by the United States Patent and Trademark Office between 1976 and 2015 are novel data contributed by Triulzi et al. (2020) [1]. Here we organize, describe and make available the collection of data in its entirety. This allows anyone interested to replicate the study or use the method to estimate the improvement rate of a given technology for which patents can be identified. For a detailed description of the data and methods see Triulzi et al. (2020) [1].

## Specifications Table

**Table:** 

Subject	Management of Technology and Innovation, Strategy and Management
Specific subject area	Technological Change, Performance Curves, Technology Development, Technology Dynamics
Type of data	Tables Figures Comma separated value files
How data were acquired	• Time series of functional performance for 28 different technologies were acquired by Christopher L. Benson and Christopher L. Magee by looking for long series of performance data points in reputable sources (scientific articles, magazines, industry reports, etcetera) for as many technologies that could be found. The method is described in Benson (2014) [2] and Benson and Magee (2015a) [3]. • Performance data for Magnetic Materials were acquired by Subarna Basnet and described in Basnet (2016) [4]. • Performance data for Hybrid Corn were collected by Maryam Barry, Giorgio Triulzi and Christopher L. Magee by analysing multiple sources: patent data where field trials were described and yields data from different US states. The collection method is described at length in Barry et al. (2017) [5]. • Patent sets for 28 of the 30 domains were collected by Christopher L. Benson and Christopher L. Magee by applying a novel Classification Overlapping Method described in Benson and Magee (2013) [6] and (2015b) [7]. • The patent set for Magnetic Materials was collected by Subarna Basnet through an application of the same method. The process is described in Basnet (2016) [4]. • The patent set for Hybrid Corn was collected by Maryam Barry, Giorgio Triulzi and Christopher L. Magee through a combined used of patent classes and keywords. The method is described in Barry et al. (2017) [5]. • Raw data on patent information and citation relationships for 5.259.906 patents granted by the United States Patent and Trademark Office (USPTO) between 1976 and 2015 were downloaded from patentsview.org. • Normalized and unnormalized patent-based measures, such as patent centrality, number of citations received or the age of the cited patents, that are tested as predictors of the improvement rate were calculated following the methodology described in Triulzi et al. (2020) [1].
Data format	• Raw (performance time-series) • Processed (patent-based indicators) • Analyzed (empirical technology yearly improvement rates and estimated ones based on patent-data)
Parameters for data collection	• Data on performance time series were collected from various sources (scientific articles, specialized websites, industrial magazines or reports) according to the criteria of availability of long time series and credibility of the source. •Patent data only includes utility patents granted by the USPTO between 1976 and 2015.
Description of data collection	• Performance data was copied or downloaded from the sources described in the section “How data were acquired” of this table. • Patent data was downloaded by using two different platforms Patsnap (for retrieving patent numbers according to queries that respected the COM method described in Benson and Magee (2013 [6] and 2015b [7]) and Patentsview (to download data used to compute the different variables described in this article). • Patent-based measures for 5.259.906 US granted utility patents and their average value for each of the 30 domains were computed as described in the related article by Triulzi et al. (2020).
Data accessibility	Repository name: Mendeley Data Data identification number: DOI 10.17632/f4fj887y67.1 Direct URL to data: http://dx.doi.org/10.17632/f4fj887y67.1
Related research article	The data in this Data in Brief article has been used to test different patent-based predictors of the improvement rate for 30 different technologies. This research is described in the following article: Triulzi G., Alstott, J., Magee, C.L., “Estimating Technology Performance Improvement Rates by Mining Patent Data”, Technological Forecasting and Social Change, 158(September) (2020) 120100.

## Value of the Data

• This data offers an empirical view of technology yearly improvement rates (TIRs) and validates Moore's law relevance for 30 different technologies. Furthermore, it provides carefully selected patent sets for 30 technology domains and a variety of indicators that can be used to predict technologies’ improvement rate or for empirical studies of technology evolution along defined technological trajectories.
• This data is of interest to scholars and practitioners studying technological change. It can also be used for pedagogical purposes in courses on technology monitoring and intelligence and econometrics of technological change.
• Patent centrality measurements for 5.259.906 USPTO granted patents can be used to estimate TIRs for technologies for which we do not have reliable time series of their performance evolution, following the method described in Triulzi et al. (2020) [1].
• The data can also be used to test alternatives predictors of the technology improvement rate, using those tested in the papers Triulzi et al (2020) [1] and Benson and Magee (2015a) [3] as benchmarks for predicting power. It can also be used to analyse the internal structure of relationships between inventions, inventors or assignees within a domain over time or across domains.
• The added value of the patent data provided, compared to patents retrieved for given technology classes (using patent classification systems such as the International Patent Classification or the Cooperative Patent Classification) is that our data is grouped in technology domains, whose definition includes artefacts that achieve the same function *and* use the same scientific principles, as opposed to commonly used classification systems that only rely on one of the two definitions.

## 1 Data Description

All .csv and .xlsx files described in this paper are available in Mendeley Data repository at http://dx.doi.org/10.17632/f4fj887y67.1 [8].

### 1.1 Performance time series

In Table 1, we summarize the information on the data used to empirically measure the TIR for 30 technologies. For each of these technologies, the table describes how many data points the performance time-series has and its year range, as well as the performance variable that describes the time-series. We also report the data source of each time series as the paper in which it was first used. In that paper the reader can find more information on how each one was collected. The time series are available in the file “performance_time_series.csv”, which contains 398 performance observations in total, for all 30 domains over time and five columns (Year, Data, Domain, Metric and Units).

**Table 1:** Summary information for the technology domains’ performance variables.

Domain ID name	Domain complete name	Performance variable	Variable unit	Data source	# of data points	1st year	Last year
3D PRINTING	Industrial stereolitography	printing speed and build volume over layer thickness and machine size and cost	Speed * build volume/(layer thickness *machine size*cost)	B&M (2015A)	5	1991	2006
AIRCRAFT	Aircraft passenger transportation	passenger transported per mile per hour	Passengers*miles/hour	B&M (2015A)	12	1926	1975
BATTERIES	Electrochemical battery energy storage	amount of energy stored per kilogram	wh/kg	B&M (2015A)	13	1970	2004
CAMERA	Camera sensitivity	mv micro per squared meter	mV/m^2	B&M (2015A)	11	1987	2008
CAPACITOR	Capacitor energy storage	amount of energy stored per kilogram	Wh/kg	B&M (2015A)	9	1970	2005
COMB ENGINE	Combustion engines	amount of energy produced per weight of engine	W/kg	B&M (2015A)	24	1896	2002
CT	Computed Tomography	CT scan resolution over scan time	1/(mm*scan time)	B&M (2015A)	13	1971	2006
ELECTRIC COMPUTATION	Electronic computation	electronic computations per second	cps	B&M (2015A)	19	1943	2007
ELECTRIC MOTOR	Electric motors	Power of electric motor per kg	W/kg	B&M (2015A)	11	1881	1993
ELECTRIC TELECOM	Electrical information transmission	Kilobyte of information transmitted per dollar spent	kbps / Million $	B&M (2015A)	10	1858	1983
ELECTRO POWERTRANS	Electrical energy transmission	AC electricity transmission powered distance	W*Km	B&M (2015A)	10	1889	1983
FLYWHEEL	Flywheel energy storage	energy per weight	kwh/kg	B&M (2015A)	7	1975	2003
FUELCELL	Fuelcell energy production	amount of energy produced per dollar spent	kW/$	B&M (2015A)	5	1970	1996
GENOME	Genome sequencing	sequenced base pairs of genome per dollar spent	BP/$	B&M (2015A)	7	1970	2004
HYBRID CORN	Hybrid corn productivity	productivity of hybrid corn varieties per acre cultivated	Bushel per acre	Barry et al. (2017)	20	1996	2015
IC	Integrated circuit processors	number of transistors per die in microprocessors	transistors / die	B&M (2015A)	12	1972	2006
INCANDESCENT	Incandescent artificial illumination	quantity of visible light emitted per dollar spent	1000 lumenhour/$	B&M (2015A)	9	1883	1990
LED	LED artificial illumination	quantity of visible light emitted per lamp	lumen/lamp	B&M (2015A)	15	1972	2009
MAGNETIC INFO STORAGE	Magnetic information storage	magnetic memory hard disk mbits per cc	mbits/cc	B&M (2015A)	23	1970	2003
MAGNETIC MAT	Permanent magnetic materials	amount of energy stored per volume	KJ / m^3	Basnet (2016)	18	1917	2008
MILLING	Milling machines	horse power over accuracy	average HP/total accuracy in mm	B&M (2015A)	6	1939	2012
MRI	Magnetic Resonance Imaging	resolution per time per dollar spent of magnetic resonance imaging	1/(mm*sec*$)	B&M (2015A)	6	1980	2006
OPTICAL INFO STORAGE	Optical information storage	amount of memory per cc	Mbits/cc	B&M (2015A)	15	1981	2004
OPTICAL TELECOM	Optical Information Transmission	optical telecommunication bandwidth per length over cost	kbps*km / Million $	B&M (2015A)	13	1988	2002
PHOTOLITHOGRA-PHY	Photolitography	areal throughput over accuracy	squared inches / hr*micron	B&M (2015A)	11	1962	1986
SEMICOND INFO STORAGE	Integrated circuits information storage	number of transistors per die in memories	transistors / die	B&M (2015A)	20	1959	2007
SOLAR PV	Solar photovoltaic energy storage	amount of energy stored per dollar spent	Watts / $	B&M (2015A)	35	1968	2009
SUPERCONDUCTOR	Superconductivity	critical temperature	1/ deg K	B&M (2015A)	7	1970	1995
WIND	Wind turbine energy generation	amount of energy generated per dollar spent	W/$	B&M (2015A)	8	1970	2011
WIRELESS TELECOM	Wireless information transmission	throughput	Kbps	B&M (2015A)	15	1970	2009

Note: B&M (2015) stands for Benson and Magee (2015a)[3]

**Fig. 1:**
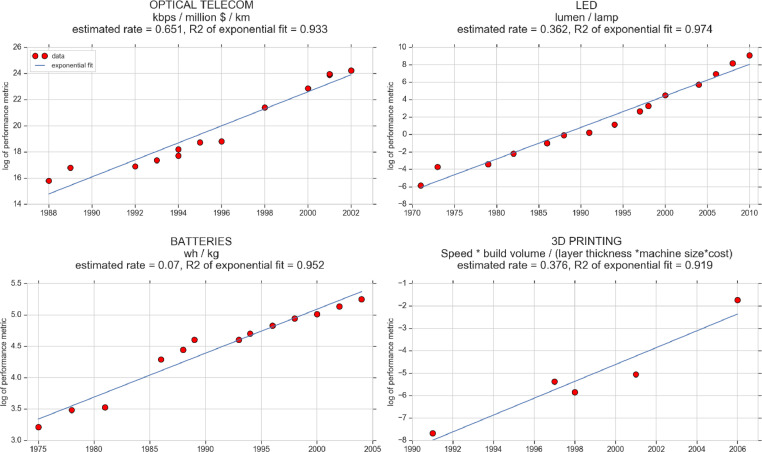
log-linear plot for four performance time series.

Fig. 1 shows, using four examples, how the empirical TIR was estimated using the time series described in Table 1. Log-linear plots of the performance variable against time were made and a linear fit of the data was performed. The slope of the line is the TIR (which correspond to the rate variable of an exponential curve). As explained by Benson (2014) [2] and Benson and Magee (2015a) [3], the estimation of the empirical TIR (second column of Table 2), is obtained by looking only at record-breakers and, when possible if the time series was long, only post-1976 data points, to match them with the period for which patent data is available. However, in the file ‘performance_time_series.csv” we make all data points available.

**Table 2:** Empirical TIR and estimated one based on patent variable ‘meanSPNPcited_1year_before_randomized_zscore_RPbyYear’.

Domain ID name	TIR	TIR *R*^2^	Estimated TIR from patent data
3D printing	0.376	0.92	0.516
aircraft	0.122	0.98	0.059
batteries	0.07	0.95	0.121
camera	0.156	0.99	0.341
capacitor	0.146	0.97	0.088
comb engine	0.057	0.82	0.124
ct	0.367	0.78	0.223
electric computation	0.33	0.9	1.020
electric motor	0.031	0.84	0.077
electric telecom	0.143	0.9	0.177
electro powertrans	0.149	0.92	0.205
flywheel	0.09	0.92	0.118
fuelcell	0.144	0.99	0.214
genome	0.293	0.91	0.124
hybrid corn	0.012	0.8	0.048
ic	0.363	0.97	0.436
incandescent	0.045	0.93	0.101
led	0.362	0.97	0.281
magnetic info storage	0.319	0.88	0.234
magnetic mat	0.048	0.96	0.179
milling	0.034	0.96	0.042
mri	0.475	0.88	0.343
optical info storage	0.271	0.95	0.403
optical telecom	0.651	0.93	0.375
photolithography	0.24	0.85	0.185
semiconductor info storage	0.432	0.98	0.454
solar pv	0.095	0.94	0.161
superconductor	0.095	0.73	0.113
wind	0.092	0.93	0.066
wireless telecom	0.504	0.86	0.425

Table 2 reports the empirical TIRs for each technology domain, obtained as shown in Fig. 1, the R^2^ of the linear fit on a log-linear plane, as a measure of the goodness of fit of the exponential hypothesis, and the estimated TIR coming from patent data. The latter is obtained using the method briefly summarized in Section 3.2 of this document and explained at length in Triulzi et al. (2020) [1].

**Fig. 2:**
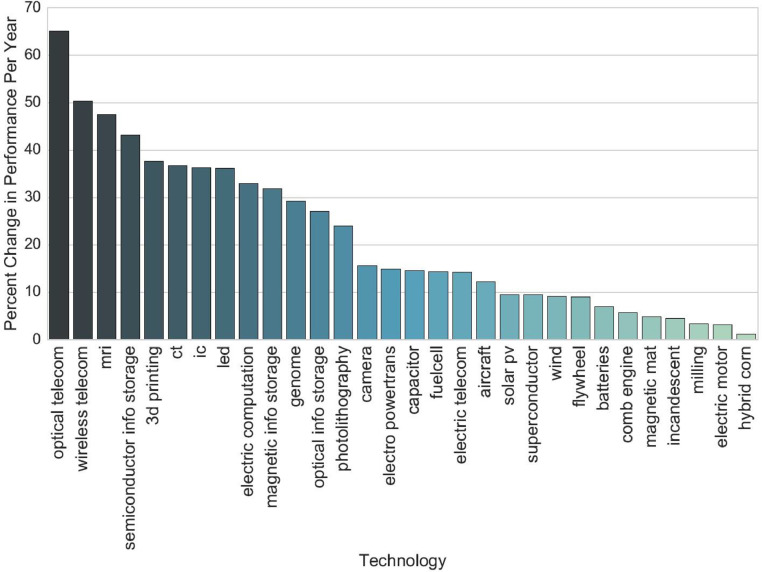
Ranking of technology domains sorted by fastest improvement rate.

Fig. 2 shows a bar plot of the empirically observed improvement rate for the 30 technology domains (using the second column of Table 2).

### 1.2 Patent data

Table 3 contains a variable dictionary for the data included in the file “Domains_patent_info.csv” (i.e. the description of the content of each column of the file). The file contains information on different variables computed for USPTO granted patents belonging to the 30 technology domains. It has one record per patent (511.570 records in total). The file “All_patents_info.csv” includes the exact same variables listed in Table 3 for 5.259.906 USPTO utility patents granted between 1976 and 2015, except for the domain information (i.e. the first raw of Table 3 does not apply).

**Table 3:** Variable dictionary for patent data file.

Variable name	Explanation
Domain	Name of the technology domain to which the patent belongs
patent_number	Patent number for US granted patents
grant_date	grant date of the patent
filing_date	filing date of the patent
filing_year	filing year of the patent
filing_year_month	filing month and year of the patent (the day is always forced to be 1). This variable is used to compute the number of months between two patents
mainclass_id	main USPC class assigned to the patent
cit_received_dec2015	number of citations received by December 2015
CIT_DEC2015_RANK_PERC_BY_YEAR	number of citations received by December 2015 normalized as a rank percentile compared to patents filed in the same year
CITE3	number of citations received within 3 years from filing
CITE3_RANK_PERC_BY_YEAR	number of citations received within 3 years from filing normalized as a rank percentile compared to patents filed in the same year
CITE3_RANK_PERC_BY_YEAR_AND_CLASS	number of citations received within 3 years from filing normalized as a rank percentile compared to patents filed in the same year and having the same USPC main class
mean_age_cited_patents	mean age of the patents cited by the focal patent, measured as difference in filing years
mean_age_cited_patents_RANK_PERC_BY_YEAR	mean age of the patents cited by the focal patent, measured as difference in filing years, normalized as a rank percentile compared to patents filed in the same year
IPC4	IPC main class of the patent
SPNP_count_2015	raw Search Path Node Pair (SPNP) centrality value as per December 2015
SPNP_count_t+2	raw Search Path Node Pair (SPNP) centrality value measured 2 years after filing
SPNP_count_t+3	raw Search Path Node Pair (SPNP) centrality value measured 3 years after filing
meanSPNPcited_1year_before	average raw Search Path Node Pair (SPNP) centrality value of the patents cited by the focal patent
SPNP_count_2015_randomized_percentile	Search Path Node Pair (SPNP) centrality value as per December 2015, normalized as a rank percentile compared to 1000 randomization
SPNP_count_t3_randomized_percentile	Search Path Node Pair (SPNP) centrality value measured 3 years after filing, normalized as a rank percentile compared to 1000 randomization
meanSPNPcited_1year_before_randomized_percentile	average Search Path Node Pair (SPNP) centrality value of the patents cited by the focal patent, normalized as a rank percentile compared to 1000 randomization
count_citations_made	number of citations made by the patent
log_count_citations_made	log of the number of citations made by the patent
within_USPCclass_citation_count	number of citations made by the patent that go to patents in the same USPC class
within_USPCclass_citation_share	share of citations made by the patent that go to patents in the same USPC class
within_IPCclass_citation_count	number of citations made by the patent that go to patents in the same IPC class
within_IPCclass_citation_share	share of citations made by the patent that go to patents in the same IPC class
within_domain_citation_count	number of citations made by the patent that go to patents in the same technology domain (N.A. for most patents as we have no information on their domain)
within_domain_citation_share	share of citations made by the patent that go to patents in the same technology domain (N.A. for most patents as we have no information on their domain)
SPNP_count_2015_RankPerc_by_year	Search Path Node Pair (SPNP) centrality value as per December 2015, normalized as rank percentile compared to patents filed in the same year
SPNP_count_t+2_RankPerc_by_year	Search Path Node Pair (SPNP) centrality value measured 2 years after filing, normalized as rank percentile compared to patents filed in the same year
SPNP_count_t3_RankPerc_by_year	Search Path Node Pair (SPNP) centrality value measured 3 years after filing, normalized as rank percentile compared to patents filed in the same year
meanSPNPcited_1year_before_RankPerc_by_year	average raw Search Path Node Pair (SPNP) centrality value of the patents cited by the focal patent, normalized as rank percentile compared to patents filed in the same year
log_meanSPNPcited_1y_before	log of the average raw Search Path Node Pair (SPNP) centrality value of the patents cited by the focal patent
SPNP_count_2015_randomized_zscore	Search Path Node Pair (SPNP) centrality value as per December 2015, normalized as a z-score compared to 1000 randomization
meanSPNPcited_1year_before_randomized_zscore	average Search Path Node Pair (SPNP) centrality value of the patents cited by the focal patent, normalized as z-score compared to 1000 randomization
SPNP_count_t2_randomized_zscore	Search Path Node Pair (SPNP) centrality value measured 2 years after filing, normalized as z-score compared to 1000 randomization
SPNP_count_t3_randomized_zscore	Search Path Node Pair (SPNP) centrality value measured 3 years after filing, normalized as z-score compared to 1000 randomization
SPNP_count_t5_randomized_zscore	Search Path Node Pair (SPNP) centrality value measured 5 years after filing, normalized as z-score compared to 1000 randomization
SPNP_count_t8_randomized_zscore	Search Path Node Pair (SPNP) centrality value measured 8 years after filing, normalized as z-score compared to 1000 randomization
SPNP_count_2015_randomized_zscore_RPbyYear	Search Path Node Pair (SPNP) centrality value as per December 2015, normalized as rank percentile of the z-score value generated by the randomization process compared to patents filed in the same year
SPNP_count_t2_randomized_zscore_RPbyYear	Search Path Node Pair (SPNP) centrality value measured 2 years after filing, normalized as rank percentile of the z-score value generated by the randomization process compared to patents filed in the same year
SPNP_count_t3_randomized_zscore_RPbyYear	Search Path Node Pair (SPNP) centrality value measured 3 years after filing, normalized as rank percentile of the z-score value generated by the randomization process compared to patents filed in the same year
SPNP_count_t5_randomized_zscore_RPbyYear	Search Path Node Pair (SPNP) centrality value measured 5 years after filing, normalized as rank percentile of the z-score value generated by the randomization process compared to patents filed in the same year
SPNP_count_t8_randomized_zscore_RPbyYear	Search Path Node Pair (SPNP) centrality value measured 8 years after filing, normalized as rank percentile of the z-score value generated by the randomization process compared to patents filed in the same year
meanSPNPcited_1year_before_randomized_zscore_RPbyYear	average Search Path Node Pair (SPNP) Centrality value of the patents cited by the focal patent, normalized as rank percentile of the z-score value generated by the randomization process compared to patents filed in the same year
bwd_self_cit	number of citations made by the patent that were directed to patents assigned to the same organization (harmonized assignee name must have exact same spelling)
share_bwd_self_cit	share of total number of citations made by the patent that were directed to patents assigned to the same organization (harmonized assignee name must have exact same spelling)
CITE3_SELF	number of citations received within 3 years from filing from other patents that have the same assignee (harmonized assignee name must have exact same spelling)
CITE3_SHARE_SELF	share of total number of citations received within 3 years that come from other patents that have the same assignee (harmonized assignee name must have exact same spelling)
count_citations_made_RANK_PERC_BY_YEAR	total number of backwards citations made normalized as a rank percentile compared to patents filed in the same year
CITE3byOthers	number of citations received within 3 years from filing from patents that have a different assignee from the one of the focal patent
CITE3byOthers_RANK_PERC_BY_YEAR	number of citations received within 3 years from filing from patents that have a different assignee from the one of the focal patent, normalized as a rank percentile compared to patents filed in the same year

Table 4 reports the mean values for a series of SPNP centrality-based patent variables computed for patents in each technology domain: the average centrality of the patents cited by patents in the domain, the centrality of the domain's patents measured after three years from filing and their centrality in 2015. All three are normalized in two different ways, one through the randomization of the entire USPTO patent citation network and the other by taking the rank percentile of the value for each patent, compared to other patents granted in the same year. These two normalization methods and their advantages and disadvantages are discussed at length in Triulzi et al. (2020) [1]. Data in Table 4, as it is presented, is available in the file “DF_means_centrality.xlsx”. The file “DF_means_all_variables.xlsx” makes available means by domains for each variable described in Table 3. It has 30 rows, one per domain, and 48 columns including the mean values for each of the variables in the rows of Table 3.

**Table 4:** Average normalized centrality variables for each domain.

Domain	meanSPNPcited_1year_before_randomized_zscore_RPbyYear	meanSPNPcited_1year_before_RankPerc_by_year	SPNP_count_t3_randomized_zscore_RPbyYear	SPNP_count_t3_RankPerc_by_year	SPNP_count_2015_randomized_zscore_RPbyYear	SPNP_count_2015_RankPerc_by_year
3D printing	0.675	0.662	0.618	0.609	0.501	0.593
aircraft	0.321	0.240	0.356	0.328	0.424	0.355
batteries	0.441	0.394	0.431	0.400	0.462	0.408
camera	0.609	0.633	0.584	0.631	0.520	0.625
capacitor	0.388	0.415	0.373	0.455	0.395	0.500
comb engine	0.444	0.523	0.490	0.562	0.498	0.543
ct	0.540	0.487	0.525	0.493	0.512	0.530
electric computation	0.782	0.826	0.763	0.754	0.624	0.721
electric motor	0.365	0.359	0.381	0.413	0.401	0.432
electric telecom	0.503	0.493	0.486	0.515	0.504	0.579
electro powertrans	0.527	0.551	0.498	0.550	0.524	0.609
flywheel	0.436	0.435	0.443	0.505	0.391	0.452
fuelcell	0.534	0.439	0.499	0.398	0.521	0.403
genome	0.445	0.315	0.380	0.305	0.428	0.298
hybrid corn	0.286	0.078	0.173	0.154	0.186	0.164
ic	0.648	0.692	0.639	0.660	0.590	0.660
incandescent	0.410	0.331	0.443	0.380	0.497	0.377
led	0.578	0.539	0.539	0.516	0.536	0.531
magnetic info storage	0.549	0.599	0.506	0.601	0.497	0.620
magnetic mat	0.504	0.466	0.478	0.452	0.469	0.433
milling	0.265	0.246	0.286	0.316	0.358	0.330
mri	0.610	0.656	0.589	0.651	0.495	0.671
optical info storage	0.636	0.668	0.633	0.623	0.572	0.571
optical telecom	0.624	0.714	0.643	0.685	0.589	0.677
photolithography	0.511	0.501	0.501	0.495	0.512	0.483
semiconductor info storage	0.655	0.659	0.632	0.639	0.571	0.668
solar pv	0.488	0.527	0.502	0.502	0.492	0.532
superconductor	0.429	0.386	0.446	0.417	0.491	0.394
wind	0.339	0.349	0.364	0.461	0.371	0.467
wireless telecom	0.644	0.713	0.629	0.680	0.614	0.700

**Fig. 3:**
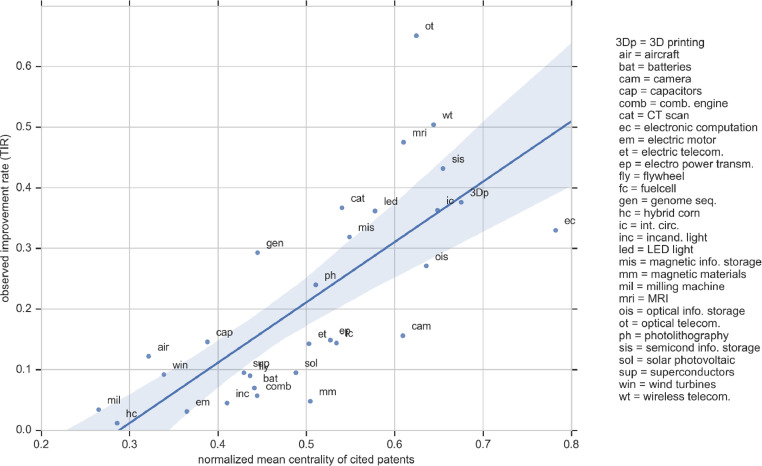
Scatter plot of a domain's mean centrality of cited patents vs. its observed improvement rate.

Fig. 3 shows the scatter plot of the observed improvement rate for each domain (the second column in Table 2) against the domain's mean centrality of the patents cited by the domain's patents (the second column in Table 4). The figure clearly highlights the strength of the relationship, which is used by Triulzi et al. (2020) [1] to train a regression that can estimate the improvement rate of any technology domain for which a reliable set of patents can be identified.

**Fig. 4:**
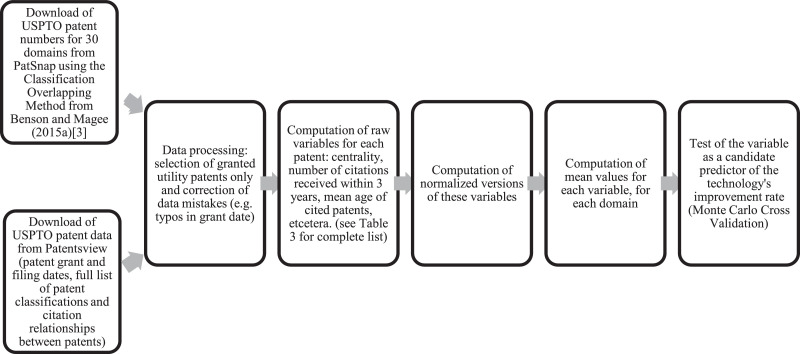
Data processing and analysis flowchart.

Finally, Fig. 4 shows the data processing and analysis flowchart, to help visualized the process followed, which is described in Section 3.1.

## 2 Experimental Design, Materials, and Methods

Patent sets for the 30 technology domains were used to compute several patent variables, which, in turn, were tested as predictors of the technology yearly improvement rate (TIR). Each variable was computed in its raw form and in a normalized form. They were then included as independent variables in a regression that estimated TIRs. The full description of the methods used can be found in Triulzi et al. (2020) [1]. Here, we report a synthesis of it.

### 2.1 Calculation of patent variables

Fig. 4 summarizes the process followed to create the datasets and process the information. For 28 of the 30 technology domains, we used patent sets provided by Benson and Magee (2015a) [3], which they retrieved using the Classification-Overlapping Method (COM) described in Benson and Magee (2013 [6] and 2015b [7]). The list of patents belonging to Magnetic Materials was provided by Basnet (2016) [4], and the one for Hybrid Corn was retrieved by Barry et al. (2017) [5]. Patent identifiers (i.e. grant number) for these 30 sets were retrieved from Patsnap (https://www.patsnap.com/). Then, basic information on their filing and grant year, classifications and their citations (made and received) were downloaded from Patentsview (https://www.patentsview.org). We then removed from the list re-issued patents, applications and non-utility patents. After that, we computed raw and normalized versions of the variables described in Table 3 and tested a subset of them, based on a theoretical selection, as candidate predictors of TIRs through a Monte Carlo cross-validation (MCCV) exercise (see next section). The subset is described in Triulzi et al. (2020) [1]. Here, we make *all* the variables computed publicly available, in case users would like to experiment with them.

### 2.2 Estimation of improvement rate

For each variable included in the file “Domains_patent_info.csv”, for each technology domain, we computed the mean value including only patents granted up to that year. We then performed a Monte Carlo Cross-Validation exercise in which we sampled randomly half of the 30 domains (creating a training set), trained a regression with that single variable as predictor of the improvement rate and then tested the performance of the regression to predict the improvement rate for the testing set of the remaining half of the domains. We did this for all years up to 2015. This exercise allow determining that two centrality variables were the predictors that ensured the most accurate estimation of the improvement rate and the least reliable on the domains included in the training test or the period of time for which the mean patent variables were computed. Finally, we estimated the full regression coefficients when including all data at disposal (i.e. all domains and patents from 1976 to 2015) and selecting the best predictor only. That regression, combined with data in the file “All_patents_info.csv”, can then be used to estimate the improvement rate for technology domains for which we only have patent data and no empirical observation of their functional performance. The estimating equation and its coefficients can be found in Triulzi et al. (2020) [1].

## Declaration of Competing Interest

The authors declare that they have no known competing financial interests or personal relationships which have, or could be perceived to have, influenced the work reported in this article.
